# Ultrathin Gas Permeable Oxide Membranes for Chemical Sensing: Nanoporous Ta_2_O_5_ Test Study

**DOI:** 10.3390/ma8105333

**Published:** 2015-09-25

**Authors:** Alexander Imbault, Yue Wang, Peter Kruse, Evgheni Strelcov, Elisabetta Comini, Giorgio Sberveglieri, Andrei Kolmakov

**Affiliations:** 1 Department of Chemistry and Chemical Biology, McMaster University, 1280 Main Street West, Hamilton, ON L8S 4M1, Canada; alexander.imbault@mail.utoronto.ca (A.I.); stephen.yue.wang@gmail.com (Y.W.); pkruse@mcmaster.ca (P.K.); 2 Oak Ridge National Laboratory, Institute for Functional Imaging of Materials and Center for Nanophase Materials Sciences, Oak Ridge, TN 37831, USA; strelcove@ornl.gov; 3 SENSOR Laboratory, Department of Information Engineering, Brescia University and CNR-INO, Brescia 25133, Italy; elisabetta.comini@unibs.it (E.C.); giorgio.sberveglieri@ing.unibs.it (G.S.); 4 Department of Physics, Southern Illinois University at Carbondale, Carbondale, IL 62901, USA

**Keywords:** Ta_2_O_5_, metal oxide, ultrathin membrane, gas sensor, gas permeability

## Abstract

Conductometric gas sensors made of gas permeable metal oxide ultrathin membranes can combine the functions of a selective filter, preconcentrator, and sensing element and thus can be particularly promising for the active sampling of diluted analytes. Here we report a case study of the electron transport and gas sensing properties of such a membrane made of nanoporous Ta_2_O_5_. These membranes demonstrated a noticeable chemical sensitivity toward ammonia, ethanol, and acetone at high temperatures above 400 °C. Different from traditional thin films, such gas permeable, ultrathin gas sensing elements can be made suspended enabling advanced architectures of ultrasensitive analytical systems operating at high temperatures and in harsh environments.

## 1. Introduction

Conductometric sensors based on semiconducting poly-(nano-) crystalline metal oxides currently constitute the least expensive and most popular gas sensing platform. The reduction of the grain size and/or dimensions of the metal oxide sensing element to nanoscopic (10–100 nm) level generally leads to enhanced gas sensitivity [1]. The latter fact stimulated the development and tests of a variety of single particle electronic devices and sensors [2]. However, the diffusion time (and therefore the sensor response time) of the analyte toward such a small receptor increases drastically with its dilution level and falls in the range of tens of minutes when the concentration approaches sub ppb level [3]. Therefore, there exists a fundamental limit of the size of the sensing element, beyond which its further reduction is not feasible. Recently high yield protocols of fabrication of a variety of free standing metal oxide membranes and laminates have been implemented via using sacrificial organic fillers [4], low-temperature solution processed sol-gels [5], and different electrochemical routes [6,7,8]. These quasi-two dimensional (q2D) oxide membranes have nanoscopic thickness that preserves their sensing advantages while their macroscopic lateral dimensions eliminate the aforementioned size and response time limitation of the receptor. In addition, the traditional thin film metal oxide gas sensors require high temperature for activation of their surface chemistry and therefore consume additional power for heating the supporting substrate. Different from thin films, the suspended q2D oxides do not dissipate heat into the substrate and benefit from having the smallest possible thermal mass. The latter feature opens a variety of opportunities of heat management in these devices such as ultrafast thermal ramping and ultra-small power consumption via Joule heating, reactive exothermic or laser heating. The additional important advantages of nanoporous membranes are: (a) enhanced surface area of the sheet and (b) gas permeability and facile diffusion of the analytes through/into the membrane. Of the different oxides that have been used to fabricate porous membranes, tantalum oxide stands out due to its exceptional mechanical and thermal stability [7,9]. It is being explored for potential applications as a membrane material in proton exchange membrane fuel cells [10], as a working platform of atomic switches [11,12], ReRAM cells [13], and other memristive devices [14].

Extending our preliminary report [15], in this communication, we report on fabrication/characterization protocols and tests of the electrical and gas sensing properties of conductometric sensors made of a supported Ta_2_O_5_ membrane in a wide range of temperatures and environmental conditions.

## 2. Results and Discussion

### 2.1. Electrical Characterization

**Figure 1:**
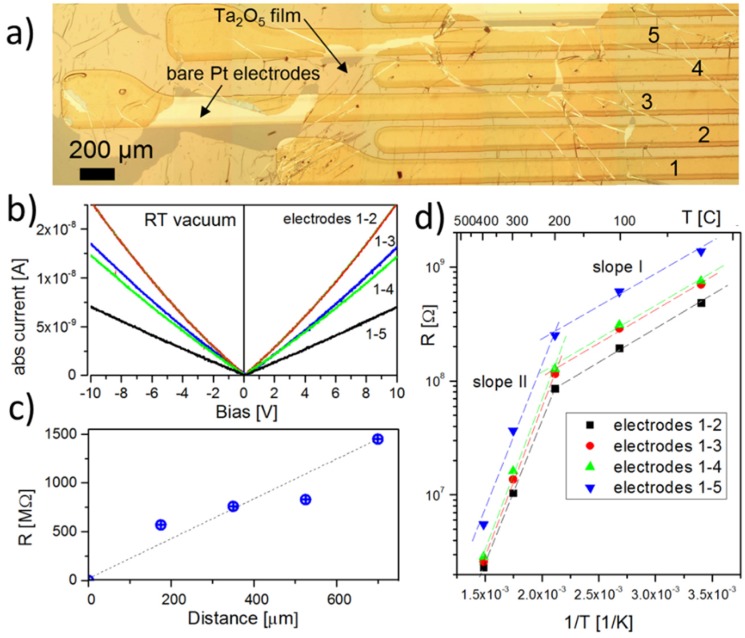
(**a**) Optical image of five Pt electrodes Lift-Off-Float-On (LOFO) coated with Ta_2_O_5_ membrane; (**b**) I–V characteristics of the Ta_2_O_5_ membrane measured between different electrodes at room temperature in vacuum. Absolute current values are shown; (**c**) Segments resistance as a function of the distance from the first electrode; (**d**) Arrhenius plot of the resistance of the Ta_2_O_5_ membrane as a function of temperature and inter-electrode distance. The straight lines are shown for eye guidance.

The membrane was placed on top of an array of parallel Pt electrodes evaporated on to SiO_2_/Si wafer (Figure 1a) using Lift-Off-Float-On or LOFO technique [7] (see Experimental Section for details). I–V characteristics of the Ta_2_O_5_ membranes appear to be nearly linear and symmetrical in the entire temperature range indicating that the Pt-membrane contacts remain ohmic (Figure 1b) and Schottky barriers at membrane-electrode interfaces do not dominate the electrical properties of the device. Comparing the slope of the I–V curves measured between different Pt electrodes (1–2, 1–3, 1–4 and 1–5 in the Figure 1a) one can conclude that the sheet resistance is increasing with inter-electrode distance and contact resistance is negligible. However, the increase of the resistance is not linear with device length (Figure 1c) presumably due to the fact that LOFO deposition onto the corrugated surface of the multi electrode chips and following annealing of the membrane lead to formation of narrow cracks or wrinkles which separate the intact domains with the average size on the order of a few hundred microns (see Figure 1a). Therefore, it is the particular morphology of the conducting path along the surface of the membrane that determines the resistance of the Ta_2_O_2_ segment between two individual Pt leads.

The Arrhenius plots of the resistance from different samples indicate the charge transport has an activation character with two characteristic temperature regions: low (room temperature to 250 ± 50 °C) and high temperature (250 ± 50 °C to 400 °C) with the activation energies of *ca.* 0.12 ± 0.05 and 0.5 ± 0.3 eV correspondingly. This implies the existence of at least two different mechanisms of conductivity in the membrane with a crossover at around 250 °C. Similar crossover was observed in many other oxides including SnO_2_ [16]. The significant error bar in the measured activation energy is due to prominent hysteretic effects upon annealing cycles. The change of the conductivity mechanism was attributed to surface transformations of pre-adsorbed oxygen and water species to chemisorbed ones: O^−^, O^2−^ or OH^−^ upon annealing at temperature above ≈220 °C (see also discussions in Reference [17]). Taking into account extremely high porosity of Ta_2_O_5_ membranes and that wet technology was used to fabricate the device it is feasible to assume that water dissociation and formation of the hydroxyl groups are mainly responsible for conductance alteration around 200 °C and apparent hysteretic effects. At very high temperatures the conductance is believed to be dominated by electron hopping via oxygen vacancies in Ta_2_O_5_ [18].

### 2.2. Gas Sensing Performance

**Figure 2:**
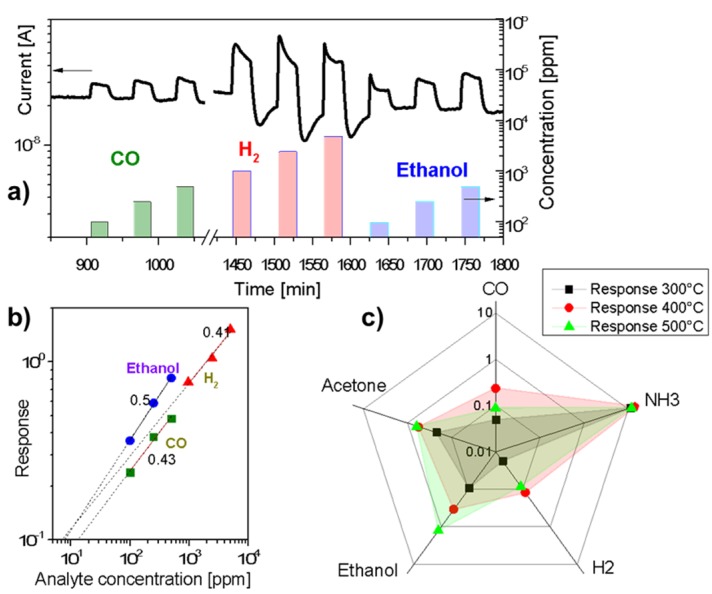
(**a**) Dynamic response of tantalum oxide membrane at a working temperature of 400 °C to pulses of carbon monoxide, hydrogen, and ethanol vapour; (**b**) Log-log plot of sensing response (Δ*I/I*_0_) of the Ta_2_O_5_ membrane to different concentrations of CO, hydrogen, and ethanol; (**c**) The response to 250 ppm of a set of analytes in air as a function of the sensor operating temperature (300 to 500 °C).

The obtained membranes manifested the ability to detect different gaseous species. As an example of the sensing behaviour towards reducing gases, Figure 2a displays the kinetic conductance responses of the membrane to pulses of carbon monoxide, hydrogen and ethanol at 400 °C. Tantalum oxide exhibits the response typical of n-type semiconductors: the exposure to reducing gases resulted in an increase of the conductance. The measured responses increased with the gas concentration. Slow drift of the base conductance of the film can be noticed. The dynamic response (Figure 2a) revealed a rapid increase of the sensor current with the introduction of carbon monoxide and ethanol (response times ≈ 1 min), whereas the recovery of air values at the end of the gas pulses was slower (*ca*. 2–3 min). The measurements of the response kinetics are limited by the chamber filling time, which is approximately 1 min. Response dynamics is almost unaffected by the operating temperature, while the recovery becomes faster at higher temperature since chemical reaction kinetic at the surface speeds up. The almost complete baseline recovery at the end of each gas pulse, observed for all of the tested analytes (except for hydrogen and ammonia), revealed a reversible interaction of the analytes with membrane surface and excluded appreciable poisoning effects. The reduced values of the base line current in the case of hydrogen and ammonia exposure are presumably due to effective reduction of the Ta_2_O_5_ membrane at 400 °C. The experimental conductance responses displayed correlation with the concentration throughout the investigated range, without any appreciable saturation (Figure 2b). The experimental sensitivity (*S* = Δ*I/I*_0_; here *I*_0_ is a base conductance) data could be well fitted by the relation typical of metal oxide semiconductor sensors: *S = a·C^B^*, where *a* is a constant characteristic of the sensing element, *C* is analyte concentration, and *B* value (0.43 for CO, 0.41 for H_2_, and 0.5 for ethanol in our case) is a function of the charge of the surface species and the stoichiometry of the involved reactions (Figure 2b). Considering a minimum appreciable response of 0.1 the detection limits for hydrogen, CO and ethanol are 7, 8 and 13 ppm correspondingly at 400 °C (Figure 2b). For reducing gases, like carbon monoxide, the sensing mechanism usually involves a chemisorption of oxygen molecules on the oxide surface with generation of active oxygen species (O_2_^−^, O^−^, O^2−^, O^−^ being the dominant and more active) by capturing electrons from the conduction band. Oxygen ionosorption at surfaces results in formation of a depletion layer decreasing the carrier concentration and making the oxide surface highly resistive. Subsequent exposure to reducing gases decreases the surface density of ionosorbed oxygen species, resulting in an increased electron concentration and current intensity. For example, when the metal oxide is exposed to CO, the gas reacts with the adsorbed oxygen species, producing CO_2_ molecules and releasing electrons into the conduction band, thus increasing the conductivity of the sample.

Figure 2c displays the conductance responses towards 250 ppm of carbon monoxide, ammonia, hydrogen, ethanol, and acetone as a function of the sensor operating temperature (300 to 500 °C). The sensitivity of the membrane toward all analytes is small at room temperature and increases progressively above 300 °C. The sensitivity to most analytes peaks at 400 °C except for ethanol and acetone, ammonia being the most reactive agent. This behaviour is consistent with the results reported on other metal oxides [19,20].

## 3. Experimental Section

### 3.1. Membrane Characterization

The tantalum sheets were prepared by electropolishing [21,22] and anodization [7,8] using 99.95% pure, 0.127 mm thick pieces of mechanically cut tantalum (Ta) foil purchased from Alfa-Aesar. H_2_SO_4_ (95%–98%, reagent grade), and HF (48%) were purchased from Fisher Scientific and mixed in a 9:1 ratio for electropolishing. For anodization, the two acids were mixed with Millipore water to yield concentrations of 2%, 4% and 6% HF in 1 M H_2_SO_4_ as indicated for each sample. The samples were first cleaned with acetone, methanol, and water and electropolished for 10 min at 15 V using a two electrode set-up with at Pt/Ir counter electrode. The same set-up with electrolytes described above was used for anodization. Electrolytes were intensively stirred during both treatments using a magnetic stir bar. The oxide sheets were scored with a scalpel and lifted off the substrate by slow immersion in water (8 mm/min) using the dipper of a Langmuir-Blodgett trough. They could then be floated onto the desired substrate using LOFO [7].

**Figure 3:**
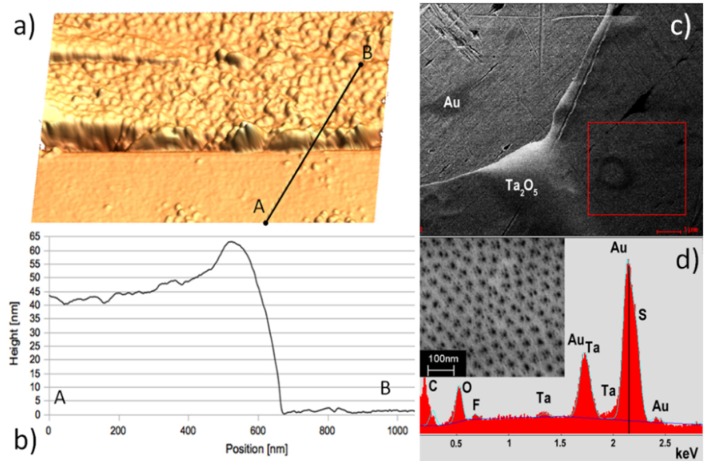
(**a**) Atomic force microscopy (AFM) image of a typical oxide sheet used to fabricate the sensors. Anodized for 10 min in 2% HF/1M H_2_SO_4_ and transferred using the LOFO technique; (**b**) AFM scan across membrane edge. Size of the image is 1.2 μm × 1.2 μm. The average thickness of the oxide sheet is measured as 45 ± 2 nm; (**c**) Scanning electron microscope (SEM) micrograph showing the uniformity of the sheets at a larger scale; (**d**) In addition to substrate (Au) and membrane (Ta, O) peaks, SEM-EDX of a sheet before measurements indicate the presence of traces of fluorine and sulfur. Inset: close-up SEM image of the pores.

To explore the morphology of the supported membranes tapping mode atomic force microscopy (AFM) was carried out using *p*-doped Si tips with a nominal radius of less than 10 nm. The thickness of the metal oxide membranes was assessed by measuring the step height at the peeled off edges using AFM (Figure 3a,b). An image of the as prepared and supported membrane (Figure 3c) was acquired on a field emission scanning electron microscope (SEM). Energy dispersive X-ray (EDX) spectra (Figure 3d) were recorded at 10 kV accelerating voltage. In addition to Ta membrane and Au substrate peaks (Figure 3d), the detected traces of fluoride and sulfur resulted from the fabrication process. The sheets are porous as previously reported and confirmed by SEM (see inset in the Figure 3d). The oxide itself is amorphous as confirmed by Raman spectroscopy [23].

### 3.2. Electrical and Gas Sensing Characterization

We used KAMINA SiO_2_/Si multi electrode chips for combinatorial electrical characterization of the membranes [24]. The membrane was placed on top of the array of parallel *ca*. 1 μm high and *ca*. 200 μm wide Pt electrodes using LOFO technique (Figure 1a and Figure 3a). The sample was slowly degassed in a high vacuum probe station before measurements. I–V measurements were conducted in a vacuum of *ca*. 10^−5^ Torr in the RT to 400 °C temperature range. At the highest tested temperature (400 °C) the leakage resistance of the SiO_2_ insulating layer was more than an order of magnitude higher compared to largest resistance of the sensing element. For the sensors fabrication, a platinum interdigitated electrode structure and a Pt heater were deposited by sputtering on the front- and back- side of 2 mm × 2 mm alumina substrates [25], respectively. These alumina transducers were calibrated using an infrared camera to correlate the power dissipated by platinum resistors with the temperature of the sample side of the alumina substrate. Similar to the aforementioned electrical setup, the membranes were deposited over the interdigitated electrodes using LOFO technique. The sensor operating temperature was achieved by applying a constant voltage to the heater, using the same Pt resistor also as a thermometer. Gas sensing properties were tested by means of the flow-through technique [25] at atmospheric pressure, using a constant synthetic air flow (0.3 L/min) as a carrier gas for the analyte dispersion. Measurements were performed in the 300 to 500 °C temperature range under a constant humidity level of 40%, in a chamber maintained at a fixed temperature of 20 °C throughout each experiment. The sensor resistance was monitored by means of the volt-amperometric technique at constant bias voltage. Herein, the sensor response *R* is defined as the relative variation in conductance upon exposure to the target gas [26] (estimated uncertainty: ±5%). The response and recovery times were calculated as previously described [27,28]. All samples were stabilized at working temperature for 10 h prior to the measurements.

## 4. Conclusions and Outlook

**Figure 4:**
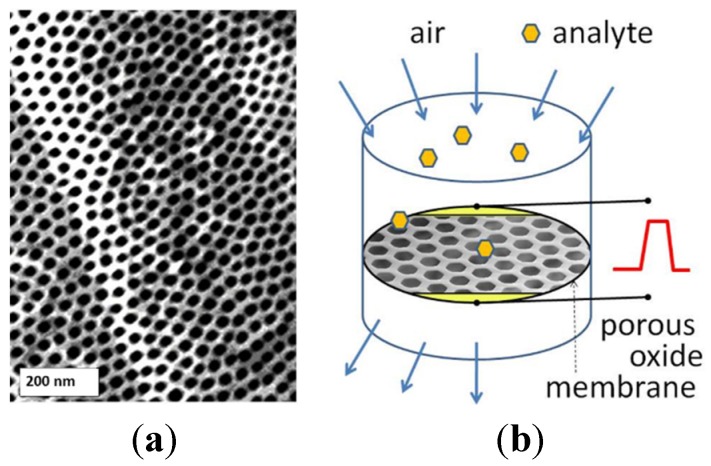
Quasi-two dimensional (q2D) porous Ta_2_O_5_ oxide membrane (**a**) and the conceptual design of the sensor with “flow-through” active sampling (**b**).

Conductometric gas sensors made of ultrathin suspended nanoporous oxide membranes can be particularly suitable for active sampling of diluted analytes. This sensing layer morphology enables a variety of advanced active sampling approaches where the environmental air containing the diluted analyte is directed onto the high surface area of a suspended chemiresistor detector assembled inside direct flow (Figure 4) or tangential flow reactor. Using this method, flows as high as few hundred milliliters of air per second can be sampled for the presence of analyte molecules by a sensing membrane 1 cm in diameter [29]. Here we reported on fabrication, conductometric measurements, and gas sensing properties of a gas sensing device with nanoporous membrane made of Ta_2_O_5_. In spite of well-known high thermal and chemical stability, Ta_2_O_5_ membranes appear to exhibit noticeable redox chemical activity at elevated temperatures above 400 °C. The use of proposed suspended architecture of the gas penetrable membranes as active sensing elements, combined with materials of superior sensing properties (SnO_2_, WO_3_, ZnO *etc.*), distributed surface functionalization and heating may lead to new designs of selective and sensitive analytical systems. In such a device the filtering, preconcentrator, and sensing elements with tailored surface chemistry and porosity can be stacked along the gas flow direction thus enhancing active sampling.
